# Analysis of Scientometric Indicators in Publications Associated with Healthy Aging in the World, Period 2011–2020

**DOI:** 10.3390/ijerph19158988

**Published:** 2022-07-23

**Authors:** Eric Rojas-Montesino, Diego Méndez, Yolanda Espinosa-Parrilla, Eduardo Fuentes, Iván Palomo

**Affiliations:** 1 Departamento de Cienciometría, Dirección de Investigación, Vicerrectoría Académica, Universidad de Talca, Talca 3460000, Chile; errojas@utalca.cl; 2 Thrombosis Research Center, Interuniversity Center for Healthy Aging, Medical Technology School, Department of Clinical Biochemistry and Immunohaematology, Faculty of Health Sciences, Universidad de Talca, Talca 3460000, Chile; diego.mendez@utalca.cl; 3 Escuela de Medicina, Universidad de Magallanes, Punta Arenas 6200000, Chile; yolanda.espinosa@umag.cl; 4 Genómica Evolutiva y Médica de Magallanes (GEMMa), Centro Asistencial, Docente y de Investigación (CADI-UMAG), Punta Arenas 6200000, Chile; 5 Interuniversity Center for Healthy Aging, Punta Arenas 6200000, Chile

**Keywords:** bibliometrics, healthy aging, older people, scopus

## Abstract

Today, the world population is aging at a fast rate. This scenario of the accelerated aging of human populations entails increased concern for healthy aging that is associated with a rise in scientific production related to the topic. In this study, the Scopus database from Elsevier was used, with a final search carried out on 5 January 2022, and various bibliometric indicators were obtained from SciVal. The study was fundamentally intended to characterize, determine trends, and understand the evolution and current state of research on the concept of “healthy aging” in the last decade. We found that there has been proportionally greater and more accelerated growth in the subject with respect to the general productivity of the world and that countries with high life expectancies tend to have made more effort to investigate this topic. The “hottest” research areas were found to be related to the cognitive aspect and the biological mechanisms involved in aging.

## 1. Introduction

Human aging manifests itself in the loss of physiological homeostasis due to progressive wear at the cellular, tissue, and organic levels, which increases the probability of the development of diseases and death [1,2]. Today, the world population is aging at a fast and sustained rate [3]. In the last six decades, the proportion of people entering the 65-years-and-over age group has been 8–10%; however, in the next four decades, it could increase to almost a quarter of the total population (22%) [3,4]. The accelerated aging of the population brings social, economic and public health problems that are difficult for most countries to solve [5]. The aging of populations is directly associated with demographic transition because of the increase in life expectancy [6,7]. Although the average global lifespan is around 72 years, there are great differences in health status and life expectancy between countries, since the burden of disease and disability at older ages is lower in developed countries [8]. This is a consequence of efforts in public health, education, nutrition and improvements in quality of life oriented towards healthy aging [7].

In the last decades, investigation into the aging process has aroused great interest in the scientific and social environments [5]. This population phenomenon is among the most important trends in 21st century America [9]. Recent research has focused on identifying the modifiable health factors that can be used during healthy aging and that reduce the burden of chronic diseases in the population to improve the quality of life of the elderly [10].

There are certain factors such as lifestyle that favor the appearance of age-related and health-related matters and that decrease life expectancy in the general population [11,12]. Aging is an accelerated phenomenon throughout the world; in 2004, there were almost 500 million people over 65 years of age, and it is expected that this number will increase to 2 billion people by 2050, which will have critical implications for planning and health provision in many countries [13]. Compared to Europe, the proportion of the elderly population in Latin America and the Caribbean has more significantly increased in recent times: in the 2000–2020 period, it increased by 57% (from 8.3% to 13.0%), while in Europe, on average, it increased by approximately 25% [14]. For Latin America, this is a great challenge due to its less developed economies and relatively low living standards [15].

In this global context, the World Health Organization (WHO) created an action plan in 2020 to encourage healthy aging in the population in the long term through the Decade of Healthy Aging (2021–2030), in which priorities and main strategies are intended to be implemented by countries to improve the physical and mental health of older persons [16]. Healthy aging is defined as the ability to maintain independence, purpose, vitality, and quality of life in old age despite unexpected medical conditions, accidents, or certain social determinants [1]. The main objective of this work was to verify the relationship between the scenario of accelerated aging of human populations and a greater concern for healthy aging associated with an increase in scientific production related to this topic. We sought to present an analysis of the bibliometric indicators of these publications to, among other things, determine the trends in the last decade in this emerging area of research, show the works with the greatest impact, determine the contributions of the countries of the world and their collaboration networks, and establish a correlation between the countries with the highest percentage of older adults and the quantity and impact of their publications. Our results can serve as a background for decision making around public policies, as well as a guide for researchers working on the topic and new researchers interested in this field.

## 2. Methodology

Bibliometric studies enable knowledge mapping and greater understanding of the state of research, trends, patterns, and hot spots in a given field of study, as well as characterizing the contribution of countries, institutions, and authors [17]. In addition, communicating these findings is important for the decision making of public management [18].

In this study, the Scopus [19] database from Elsevier [20] was used. This source was chosen because it currently offers greater coverage and better data indexing than other databases. First, publications associated with the key term “healthy aging” were obtained, and a frequency analysis of all the keywords in this dataset was carried out by selecting 15 keywords related to the topic with the most occurrences. The sample covered a decade of scientific productivity between 2011 and 2020. The search only considered citable documents (articles, reviews, conference papers, books, and book chapters), and keywords were searched for in the title, abstract, and keywords fields. In this way, the query used to create the database was: TITLE-ABS-KEY (“healthy aging” OR “healthy ageing” OR “health ageing” OR “health aging” OR “successful aging” OR “successful ageing” OR “aging well” OR “ageing well” OR “healthful aging” OR “healthful ageing” OR “healthy elderly” OR “healthy older adults” OR “healthy old age” OR “healthy longevity” OR “healthful longevity”) AND (LIMIT-TO (PUBYEAR, 2020) OR LIMIT-TO (PUBYEAR, 2019) OR LIMIT-TO (PUBYEAR, 2018) OR LIMIT-TO (PUBYEAR, 2017) OR LIMIT-TO (PUBYEAR, 2016) OR LIMIT-TO (PUBYEAR, 2015) OR LIMIT-TO (PUBYEAR, 2014) OR LIMIT-TO (PUBYEAR, 2013) OR LIMIT-TO (PUBYEAR, 2012) OR LIMIT-TO (PUBYEAR, 2011)) AND (LIMIT-TO (DOCTYPE, “ar”) OR LIMIT-TO (DOCTYPE, “re”) OR LIMIT-TO (DOCTYPE, “cp”) OR LIMIT-TO (DOCTYPE, “ch”) OR LIMIT-TO (DOCTYPE, “bk”)).

The final search was carried out on 5 January 2022, and various bibliometric indicators were also obtained from SciVal [21] on 6 January 2022. The SciVal platform uses the Scopus database as raw material and is updated monthly; the data presented here correspond to the 25 December 2021 version. The indicators related to citations considered self-citations, and the quartiles were obtained from the Scimago Journal & Country Rank [22]. The presented quartile is best associated with one or more disciplines of the journal, e.g., the “International Journal of Environmental Research and Public Health” is classified under Health, Toxicology and Mutagenesis (Q1), Pollution (Q2) and Public Health, Environmental and Occupational Health (Q2). The best quartile in this case was Q1, so it was set as the representative quartile of journals. The data were processed and analyzed using Microsoft Excel [23]. VOSviewer [24] was also used to generate relationship maps to visualize connections between the entities associated with the publications. Additionally, for each country, we obtained the latest available versions (2020) of the percentages of the population aged 65 and over and life expectancy at birth from The World Bank Group [25].

## 3. Results

### 3.1. Productivity

**Figure 1:**
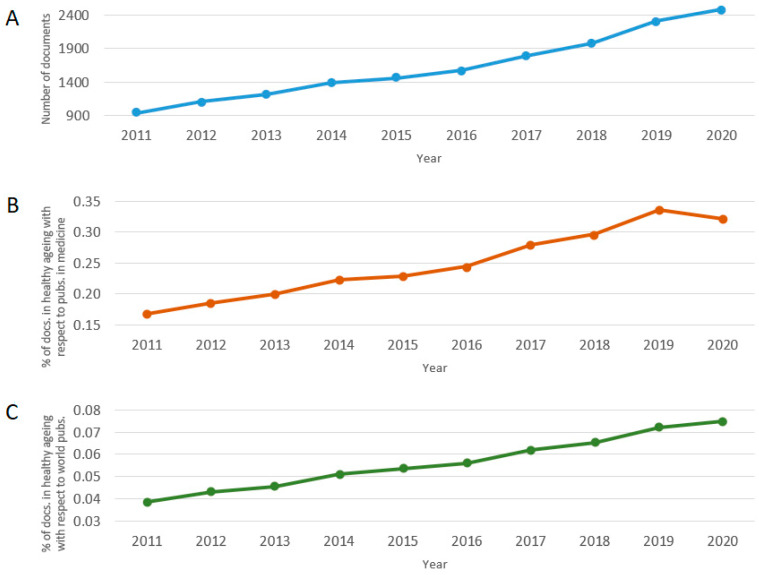
(**A**) Number of documents published per year on the topic of “healthy aging” according to the Scopus database and the percentage of documents on healthy aging with respect to (**B**) the total number of documents published in the “Medicine” area in the world and (**C**) the total number of documents published in the world during the period of 2011–2020.

**Figure 2:**
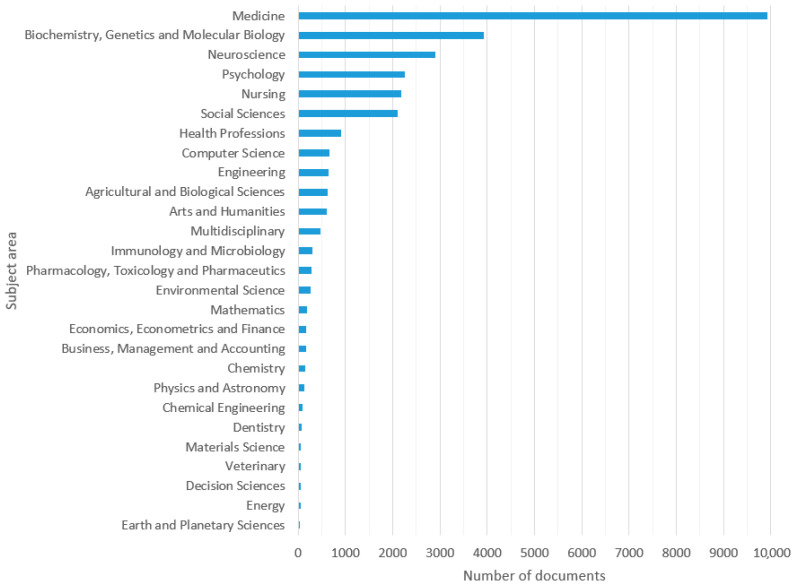
Number of published documents on the subject of “healthy aging” according to the study areas defined by Scopus (2011–2020).

In total, 16,248 publications were obtained according to the parameters established in the methodology. From these, 13,171 (81.1%) were articles, 1743 (10.7%) were reviews, 702 (4.3%) were conference papers, 551 (3.4%) were book chapters, and 81 (0.5%) were books. Most of the documents were published in English (*n* = 15,598; 96%), followed by Spanish (*n* = 174; 1.1%), Chinese (*n* = 106; 0.7%), French (*n* = 103; 0.6%) and German (*n* = 91; 0.6%). Other languages less frequently found were, in descending order of appearance, Japanese, Portuguese, Russian, Italian, Korean, Czech, Dutch, Turkish, Croatian, Polish, Hungarian, Greek, Persian, Swedish, Bosnian, Danish, Serbian, Slovak, Slovenian, Estonian, Hebrew, Norwegian and Ukrainian. Over half of the documents were published in open access journals (*n* = 9080; 55.9%) belonging to the categories of Green (*n* = 7949; 48.9%), Gold (*n* = 3825; 23.5%), Bronze (*n* = 1770, 10.9%) and Hybrid Gold (*n* = 802, 4.9%). It should be noted that documents may be classified in more than one of these categories because different publishers may use one or more of these open access options. Figure 1A shows the number of documents published per year on the topic. It can be observed that, since 2011, there has been a sustained increase in the productivity of publications on the topic. Figure 1B,C shows the percentage of publications on healthy aging with respect to the total of documents in Medicine (according to Scopus) and the general total number of documents in the world per year (same types of documents defined in the methodology). In both cases, the proportion of documents on healthy aging tended to grower higher over time, except in comparison with Medicine in 2020, probably due to the increase in research in the area as a consequence of the start of the COVID-19 pandemic. These results are consistent with the annual growth rates in the period; while the world and Medicine categories presented rates of 3.2% and 3.3%, respectively, publications on healthy aging presented a rate of 10.3%. Figure 2 shows the number of documents according to the study areas defined by Scopus, with Medicine being the discipline with the highest percentage of documents (61.1%), followed by Biochemistry, Genetics and Molecular Biology (24.2%), Neuroscience (17.9%), Psychology (13.9%), Nursing (13.4%) and Social Sciences (13.0%). It should be noted that documents may belong to more than one study area, depending on the disciplines covered by the journal in which the document was published.

**Figure 3:**
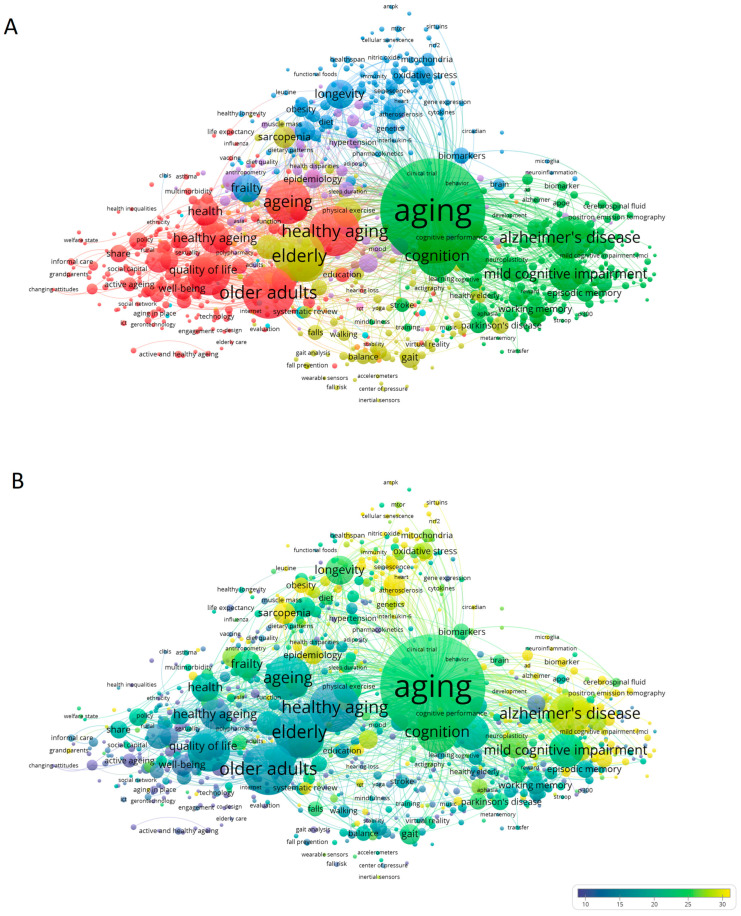
Co-occurrence map of the keywords defined by the authors and showing at least ten occurrences. The size of the circles is based on the number of occurrences, while the colors represent (**A**) the strength of the relationships and (**B**) the average number of citations. The map was built with the publications on the subject “healthy aging” according to Scopus (2011–2020).

Next, we constructed a co-occurrence map of the keywords defined by the authors that showed at least ten occurrences and found that 884 out of the 23,548 extracted terms met these criteria. At least four clusters could be easily distinguished (Figure 3A): the green cluster in Figure 3A is associated with cognition and brain aging, and it includes terms such as “Alzheimer’s disease”, “cognition”, “dementia”, “memory” and “mild cognitive impairment”; the blue cluster is associated with the biology and mechanisms of aging, and it includes terms such as “biomarkers”, “genetics”, “nutrition”, “longevity”, “obesity”, “mitochondria”, “oxidative stress” and “frailty”; the red cluster is associated with the social sphere and public health, and it includes terms such as “older adults”, “healthy aging”, “depression”, “successful aging”, “health”, “well-being” and “social support”; and the yellow cluster is associated with mobility and loss of motor skills, and it includes terms such as “elderly”, “physical activity”, “sarcopenia”, “gait”, “mobility”, “falls”, “walking”, “balance” and “rehabilitation”. Figure 3B shows the keyword co-occurrence map with emphasis on the average number of citations. Topics in colors closer to yellow received more citations and should thus be considered hotter topics. These results suggest that the scientific publications are more focused on aspects related to the cognitive aspect and biological mechanisms of healthy aging.

**Figure 4:**
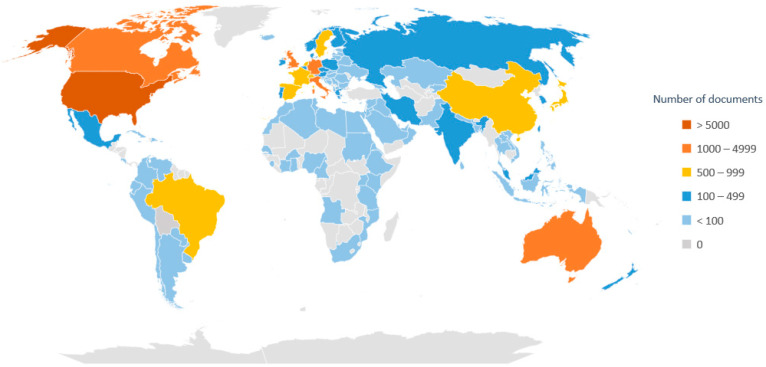
Ranges in the number of documents published by country on the subject of “healthy aging” according to Scopus (2011–2020).

Figure 4 shows the number of documents published by country divided into ranges. The United States was found to be the country with the highest production, participating in 31.7% of the publications (*n* = 5156), followed by the United Kingdom (*n* = 1916; 11.8%), Germany (*n* = 1331; 8.2%), Canada (*n* = 1242; 7.6%), Italy (*n* = 1223; 7.5%) and Australia (*n* = 1113; 6.9%). The five institutions with the highest participation were found to be the National Institute on Aging (*n* = 362), the University of California San Francisco (*n* = 346), the University of Pittsburgh (*n* = 345), the “Institut National de la Santé et de la Research Médicale–INSERM” (*n* = 259), and the Harvard Medical School (*n* = 256). All the institutions with the largest number of publications were shown to be in the United States except for the INSERM in France. The journals most chosen to publish the contents were shown to be Plos One (*n* = 322), Frontiers in Aging Neuroscience (*n* = 278), Neurobiology of Aging (*n* = 230), Journals of Gerontology Series A (*n* = 218) and Journal of Alzheimer’s Disease (*n* = 201).

**Figure 5:**
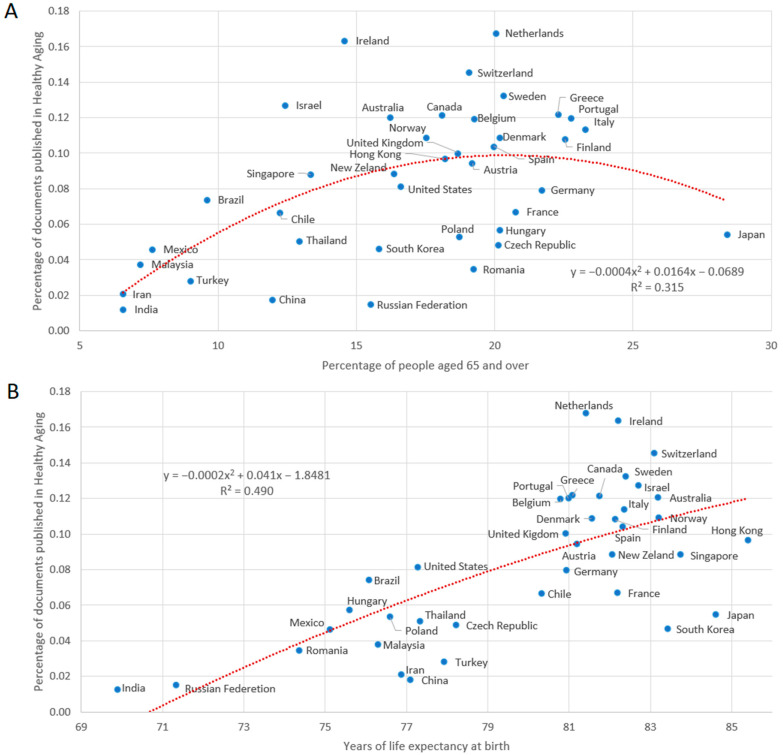
Dispersion and quadratic regression of the percentage of documents published on healthy aging with respect to the total number of published documents (citable items) by country (2011–2020) and the percentage of people aged 65 and over (**A**), as well as the life expectancy at birth (in years) (**B**). Indicators related to aging were set according to data from the World Bank Group for the year 2020. Only countries with at least 50 documents on the subject in the period were considered.

In order to normalize the contribution of the countries to the subject, we obtained the total number of publications (same period and same types of documents) for each one and calculated what percentage of this total represented research on healthy aging. This indicator measured the degree of attention that the country gave to the field of study. In Figure 5, we illustrate a correlation between research on healthy aging and two indicators related to aging—the percentage of people aged 65 and over (A) and the years of life expectancy at birth (B)—through a quadratic regression. For case A, we found that there was a greater effort to publish on the topic in countries with an elderly population of up to approximately 20%, and then the proportional number of publications began to decline. With respect to case B, we found a more direct correlation: countries with higher life expectancies tended to have higher publication rates.

### 3.2. Visibility and Impact

The 16,248 documents included in this study accumulated 325,713 citations (average of 20 citations per document). In this sense, the publications presented a field-weighted citation impact of 1.28, which implies that the publications were cited 28% above the world average in their respective areas of study, and 89.5% of the documents were cited at least once. On the other hand, 18.7% of the total productions were classified within the top 10% most cited worldwide. According to the Scimago Journal & Country Rank, 64.3% of the documents were published in journals in the first quartile (Q1), and 84.1% were published in either Q1 or Q2. The variation of the percentage of documents in Q1 fluctuated between 68.9% in 2017 and 59.8% in 2020. The five journals with the highest impact factor (SJR) were found to be *Nature Reviews Molecular Cell Biology* (SJR = 37,461), *Cell* (SJR = 26,304), *Nature Reviews Immunology* (SJR = 20,529), *New England Journal of Medicine* (SJR = 19,889) and *Nature* (25,993), all of them being Q1 journals. Table 1 shows the top ten publications with the highest number of citations received. It can be seen that the documents were relatively old, the most recent being from 2014, which is probably due to the fact that these publications had more time to accumulate citations. Finally, it is worth mentioning that 90% of these publications were open access.

**Figure 6:**
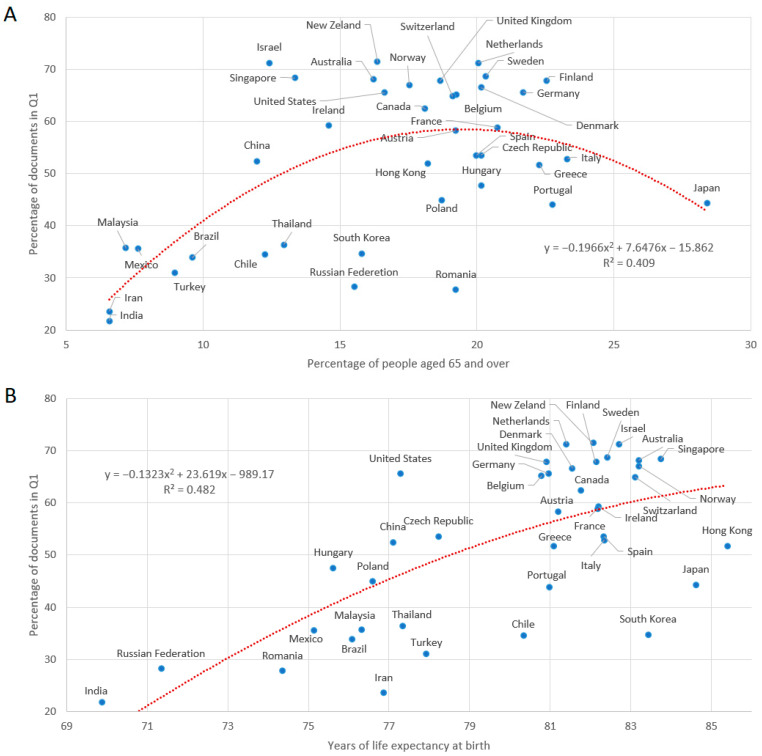
Dispersion and quadratic regression of the percentage of documents published by country in the first quality quartile (Q1) and the percentage of people aged 65 and over (**A**) and the life expectancy at birth (in years) (**B**) in the period of 2011–2020. Indicators related to aging were set according to data from the World Bank Group for the year 2020. Only countries with at least 50 documents on the subject in the period were considered.

Figure 6 shows the percentage of documents in Q1 by country and its relation to the percentage of people aged 65 and over (A) and life expectancy at birth (B). We can see that the percentage of quality publications tended to be higher in countries with an elderly population of up to 20%, and then the proportion of documents in Q1 starts to decrease; on the other hand, there seemed to be a more directly positive relationship with the life expectancies of nations. The longer the life expectancy, the better the quality of journals the documents tended to be published in. The relation between the documents classified in Q2, Q3 and Q4 and the longevity variables was inverse to that presented for Q1. 

### 3.3. Collaborations

Regarding collaborations, 28.4% of the publications were international collaborations, 39.3% were national collaborations, 25.1% were institutional collaborations, and only 7.2% were written by a single author. The assignment of the country or countries associated with each document was found to depend on the nationalities of the institutions to which their authors belonged, that is, the publications were classified by country according to their affiliations. Figure 5 shows the evolution of these types of collaboration per year in the period defined in this study (2011–2020). It can be observed that most of the works written by more than one author were the result of collaboration with researchers affiliated with the same country. In 2014, international collaboration surpassed purely institutional collaboration, while documents published by a single author remained around 10% with a downward trend. It should be noted that the types of collaboration were found to be mutually exclusive according to hierarchy: international, national, institutional, and sole authorship. For example, publications that presented international and institutional collaboration were exclusively classified as international. Figure 6 shows a map of collaboration between countries. Those that had at least two documents published on the subject were considered. As stated above, the United States was the country with the highest production, although, according to the size of its representation in the graph compared to that of other countries, it may be concluded that a large portion of its documents were not written in international collaboration. In terms of relations between countries, a clear closeness could be seen between those countries with common languages, with the blue cluster faithfully representing this fact because it mostly comprises Latin American countries in addition to Spain.

## 4. Discussion

Life expectancy and longevity have increased in recent decades, a trend that is particularly noticeable in developed countries [36]. During aging, molecular and cellular deterioration affect the physiology of tissues, which exponentially increases with age. Therefore, this process is accompanied by an increase in the burden and susceptibility of aging-related diseases and death [37,38]. It is in this context that the concept of healthy aging arises; some authors define healthy aging as the ability to maintain independence, purpose, vitality and quality of life in old age despite unexpected medical conditions, accidents, or certain social determinants [1], and other authors define healthy aging in a simpler way such as the disease-free aging process [39].

Due to the problem of accelerated aging of populations, there has been a growing increase in scientific production associated with healthy aging and related topics, which can be clearly observed in Figure 1 through the presented sustained increase in productivity, with an annual growth rate of 10.3%. Additionally, we verified that the subject has grown at a faster rate than the area of Medicine and the general productivity of the world. In addition, it is important to highlight that, according to these data, more than 80% of the publications correspond to original research works, whose objective was to generate new and robust information that will have critical implications for the planning and provision of social and health care for the elderly [13].

According to the data collected in Figure 2, the disciplines with the highest percentage of publications in the decade were Medicine (61.1%), Biochemistry, Genetics and Molecular Biology (24.2%), and Neuroscience (17.9%), which may be due to the fact that these disciplines are a fundamental part of the study of healthy aging. According to the literature, key healthy habits are nutrition, physical activity, stress management, avoiding the consumption of harmful substances, and an adequate sleep cycle that, when established at an early age and practiced throughout life, favors people’s longevity [40,41,42]. In the current demographic context (accelerated aging), the approach that nations are establishing is investing in research and the development of health policies to promote healthy aging, with the aim of reducing the burden of disease in the population and reducing future costs associated with non-healthy aging morbidity [40,41,42,43,44]. The results shown in Figure 5 are consistent with this idea because, broadly speaking, the greater longevity and life expectancy of the population are related to greater efforts in research on healthy aging. Additionally, research efforts on the subject were found to be stronger in relation to life expectancy rather than the percentage of older adults in a country. This could indicate that public and research policies are anticipating a possible future scenario. One of the main objectives of research in Biochemistry, Genetics and Molecular Biology is the identification of predictive biomarkers of disease to understand physiological aging and thus prevent or delay the onset of age-associated diseases [37]. In the case of neuroscience, investigations intended to reveal the mechanisms involved in the development of neurodegenerative diseases could lead to identifying risk factors or new treatments that could improve the quality of life of patients [45]. For example, Alzheimer’s disease (AD) will represent a great challenge for health systems, as the number of people living with dementia associated with AD could triple from 47 to 132 million by 2050, demonstrating the importance of reducing its incidence projection [46].

The data shown in Figure 2 correlate with the results observed in Figure 3A,B, since the co-occurrence of the largest keywords defined by the authors are aging, elderly, healthy aging, aging, older adults, quality of life, Alzheimer’s disease, mild cognitive impairment, health, frailty and longevity. In Figure 3B, a focus of the scientific community on the analysis of brain and the cognitive aspects of aging can be specifically observed in tandem with the biological mechanisms of healthy aging because these topics were the most cited during the period under study. As shown in these first three figures, the sustained increase in scientific production associated with healthy aging (in the aforementioned disciplines, in particular) is associated with the estimate that the population of older adults will double between now and 2050 [47], presenting great economic challenges for countries and their health systems. In addition, geriatrics and gerontology are and will be essential in promoting the health of the elderly, optimizing their well-being, and preventing disease, weakness, frailty, and death [44].

The country with the highest scientific production associated with healthy aging was found to be the United States (31.7%), followed by the United Kingdom (11.8%), Germany (8.2%), Canada (7.6%), Italy (7.5%) and Australia (6.9%), results that are directly related to each country’s development and investment in science. This trend in scientific predominance from the US and Europe has remained constant for the last 30 years, e.g., this group produced more than 98% of the articles with the most citations by the end of the 2000s [47]. This considerably contrasts with the situation observed in Latin America, as the scientific contribution of this geographical area was found to represent a low percentage in world production due to low economic investment, low institutional development, a limited number of researchers, and higher costs of reagents and equipment even though this percentage has been on the rise in the last decade [48].

The number of citations associated with healthy aging in the period under study was found to be 20 citations per document, which represents a field-weighted citation impact of 1.28 and underlines the relevance of these studies. In addition, a significant percentage (18.7%) was within the top 10% most cited worldwide, and the Scimago Journal & Country Rank revealed that 64.3% of the documents were published in Q1 journals and 84.1% were published in either Q1 or Q2 journals. These results are quite remarkable, since publishing articles in high-impact open access journals is essential for the visibility of research [49]. Additionally, the results shown in Figure 6 indicate that the higher the longevity rates of a country (especially regarding life expectancy), the greater the tendency to publish in journals of the first quartile of quality. In this sense, the contribution of excellent research, accompanied by scientific evidence and clinical epidemiological studies, is the basis for the design of public and social policies that promote quality of life as people age, allowing them to face challenge of encouraging healthy aging [50].

**Figure 7:**
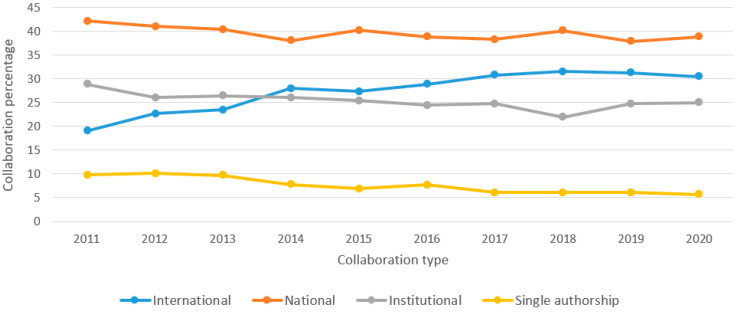
Documents on the topic of “healthy aging” with respect to the type of collaboration according to Scopus (2011–2020).

**Figure 8:**
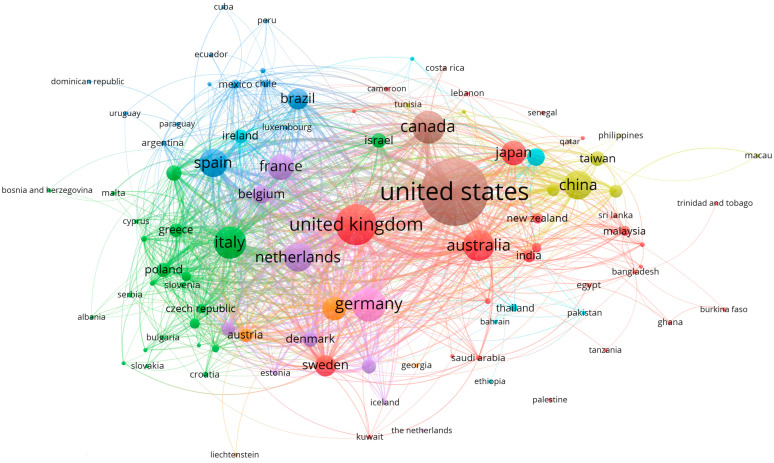
Map of international collaborations considering countries with at least two publications on the topic of “healthy aging”. Circle size is related to the number of documents. The countries are grouped by color; those with the same color have more collaborations with each other.

Figure 7 and Figure 8 illustrate the collaboration in the scientific publications analyzed in the period. Throughout the decade, the percentages of national collaboration remained generally stable, while institutional collaborations and single-author publications tended to decrease. In the case of international collaboration, there was an increasing trend until 2018, when it reached a plateau. The arrival of new technologies and greater global connectivity favored the increase in international collaboration, as it presented a series of advantages such as an increase in the number of citations, faster trial performance, extensive discussion of results, and an increasing impact factor [51]. Furthermore, there was a direct association between international collaboration and the number of citations received [52]. Figure 8 illustrates the context of collaboration among countries. It can be seen that the US had the highest production level, but most of its publications were not related to international collaborations. This may have been due to the fact that, in general, the US has the infrastructure, equipment, and qualified professionals required to carry out high-quality research without collaboration from other countries. However, US-based organizations have found that international partnerships are providing unique opportunities to further expand research and training [53]. It is common for countries in regions with less scientific development and low visibility of research, such as those in Latin America [48], to seek international collaboration to increase the impact and quality of their research.

## 5. Conclusions

The increasing rate of publications in the decade of 2011–2020 associated with healthy aging responded to the need to generate scientific knowledge that allows for solutions to be provided for the projection of accelerated aging of the population in the coming decades, as it is expected that there will be 2 billion older people by the year 2050. Research trends in healthy aging were found to mainly be associated with the study of the brain and cognitive aspects of aging, along with the biological mechanisms of healthy aging. Likewise, the results indicate nations interest in investigating healthy aging; the countries with higher percentages of aged populations and/or greater life expectancy (which can translate into the aging of their population in the future) have published more and invested more research capacity in the subject, in addition to preferring journals with high impact factors. High-quality and -impact scientific production is essential for nations to establish health policies that promote healthy aging to reduce the burden of disease and to reduce the monetary costs associated with an aging and sick population. Currently, the US and Europe are the leaders in scientific production in all areas, including disciplines focused on healthy aging. Finally, as we found a direct association between international collaboration and the number of citations received, it can be concluded that emerging countries use international collaboration with countries of greater scientific development as a tool to increase their research visibility.

Regarding the limitations of this study, we understand that the phenomenon of population aging encompasses many different facets, and this study only considered scientific publications directly related to the concept of “healthy aging” and its derivatives. Furthermore, the results of the association between indicators of aging and productivity/quality must be viewed with care. Even though common sense may indicate that aging is the cause and research on healthy aging is the consequence, our study does not formally prove this idea; instead, it simply establishes a correlation between these two factors.

For future work, we believe that it is necessary to carry out a more detailed study of the current state of research in each country and analyze more indicators that allow for a better description of the correlation between the aging of nations and their efforts in the development of healthy aging.

## Figures and Tables

**Table 1:** The top ten most cited publications on the topic of “healthy aging” according to Scopus (2011–2020) and ordered from most to least cited.

#	Authors	Title	Year	Journal	Cited by	Document Type	Open Access
1	Villeda S.A., Luo J., Mosher K.I., et al. [26]	The ageing systemic milieu negatively regulates neurogenesis and cognitive function	2011	*Nature*	1004	Article	All Open Access, Green
2	Studenski S.A., Peters K.W., Alley D.E., et al. [27]	The FNIH sarcopenia project: Rationale, study description, conference recommendations, and final estimates	2014	*Journals of Gerontology—Series A Biological Sciences and Medical Sciences*	1001	Article	All Open Access, Green
3	Deeks S.G., Lewin S.R., Havlir D.V. [28]	The end of AIDS: HIV infection as a chronic disease	2013	*The Lancet*	984	Review	All Open Access, Green
4	Börsch-Supan A., Brandt M., Hunkler C., et al. [29]	Data resource profile: The survey of health, ageing and retirement in Europe (share)	2013	*International Journal of Epidemiology*	789	Article	All Open Access, Bronze, Green
5	Pahor M., Guralnik J.M., Ambrosius W.T., et al. [30]	Effect of structured physical activity on prevention of major mobility disability in older adults: The LIFE study randomized clinical trial	2014	*JAMA—Journal of the American Medical Association*	747	Article	All Open Access, Bronze, Green
6	Powe C.E., Evans M.K., Wenger J., et al. [31]	Vitamin D-binding protein and vitamin D status of black Americans and white Americans	2013	*New England Journal of Medicine*	744	Article	All Open Access, Green
7	Martin-Montalvo A., Mercken E.M., Mitchell S.J., et al. [32]	Metformin improves healthspan and lifespan in mice	2013	*Nature Communications*	739	Article	All Open Access, Bronze, Green
8	Kanfi Y., Naiman S., Amir G., et al. [33]	The sirtuin SIRT6 regulates lifespan in male mice	2012	*Nature*	683	Article	-
9	Swanson D., Block R., Mousa S.A. [34]	Omega-3 fatty acids EPA and DHA: Health benefits throughout life	2012	*Advances in Nutrition*	654	Review	All Open Access, Bronze, Green
10	Williams A.R., Hare J.M. [35]	Mesenchymal stem cells: Biology, pathophysiology, translational findings, and therapeutic implications for cardiac disease	2011	*Circulation Research*	649	Review	All Open Access, Bronze, Green

## Data Availability

Data available in Scopus database.
